# Heart rate variability and overtraining in soccer players: A systematic review

**DOI:** 10.14814/phy2.70357

**Published:** 2025-05-22

**Authors:** Antoine Lipka, Chloé Luthardt, Teddy Tognaccioli, Beatrice Cairo, Raphael Martins de Abreu

**Affiliations:** ^1^ Department of Health LUNEX University of Applied Sciences Differdange Luxembourg; ^2^ Department of Biomedical Sciences for Health University of Milan Milan Italy; ^3^ LUNEX ASBL, Luxembourg Health & Sport Sciences Research Institute Differdange Luxembourg

**Keywords:** athletes, autonomic nervous system, football, sports medicine

## Abstract

This systematic review aims to determine if there is a correlation between heart rate variability (HRV) indices and overtraining symptoms (OTS) in soccer players. Conforming to PRISMA guidelines, a search was conducted in February 2024 on Web of Science, PubMed, EMBASE, CINAHL, and SCOPUS. Studies published in English investigating the relationship between HRV parameters and OTS in adult soccer players (>18 years) were included. Accepted study designs were randomized controlled trials, controlled clinical trials, longitudinal studies, prospective studies, cross‐sectional studies, and retrospective studies. Methodological quality was assessed using the Joanna Briggs Institute (JBI) checklist. The search identified 2041 articles, with 19 included postscreening. Most studies examined the correlation between HRV and OTS using linear indices. The average JBI checklist score was 6.3, indicating fair methodological quality. Thirteen studies showed a relationship between linear HRV parameters and OTS, linked to performance/clinical tests, training load, adaptation, fatigue, recovery, or hormonal markers. Thirteen correlations involved HRV frequency domain parameters, and 28 involved HRV time domain characteristics. HRV indexes were linked to OTS markers such as physical performance and psychological aspects in soccer players. Standardization in research methodologies, addressing confounding factors, and exploring additional indexes are crucial in the future.

## 1 INTRODUCTION

Athletes aim to optimize performance through structured and intensive training. By integrating progressive overloading with sufficient recovery, they can stimulate positive physiological adaptations, including the supercompensation effect. The supercompensation effect refers to the process by which the body temporarily adapts to training stress by strengthening physiological systems beyond their original baseline. This phenomenon occurs after the body recovers from fatigue and is primed to handle a greater workload, thereby improving athletic capacity. Effective training programs leverage the supercompensation effect by balancing exercise intensity and recovery to optimize performance gains.

However, an imbalance between training and recovery can result in stagnation or a decline in performance over a variable time frame, ranging from short‐ to long‐term. In cases where the decrease in athletic performance is followed by a supercompensation effect after recovery (<2 weeks), the term functional overreaching (FOR) is frequently used. If the athlete's performance persists or deteriorates for a period of up to 4 weeks (≤4 weeks), they may be experiencing nonfunctional overreaching (NFOR). If this condition lasts longer than 4 weeks (>4 weeks), it may indicate a more severe condition known as overtraining syndrome (OTS) (Meeusen et al., 2013).

Soccer requires a sophisticated blend of technical, biomechanical, tactical, mental, and physiological skills to achieve peak performance (Stølen et al., 2005). The sport's intermittent nature demands a frequent alternation between high and low‐intensity activities: frequent bursts of intense activity, including accelerations, decelerations, and tackles, interspersed with periods of active and passive recovery (Mirto et al., 2024). Elite players sustain a high average intensity throughout a 90‐minute match, covering roughly 10 km at 80%–90% of their maximum heart rate, aligning with the anaerobic threshold (Stølen et al., 2005). Finding the right balance is particularly concerning, as the recovering capacity from official matches and rigorous training is often seen as an essential factor for a better performance as well as injury prevention (Dellal et al., 2015). The escalating demands of modern soccer, driven by its growing commercialization and proliferation of competitions (domestic and international), have resulted in congested schedules (Dellal et al., 2015; Julian et al., 2021). Professional soccer players play, on average, three matches within 7–11 days and up to seven matches within 28–31 days in knockout phases, in addition to stressor stimulus associated with travel to and from away matches, which increases the risk of injuries and OTS (Mirto et al., 2024).

Therefore, it is necessary to keep advancing techniques for measuring the impact of training intensity, evaluating an athlete's physiological and psychological condition, and enabling individualized training programs in order to enhance performance and ensure player wellbeing. Several studies have tried to establish a scientifically evidence‐based approach to diagnose and understand the complex interplay between physiological (i.e., neural networks, intestinal microbiota, immune factors, and energy availability) and psychological factors in the different stages of overtraining but have remained inconclusive (Armstrong et al., 2021; Weakley et al., 2022). Currently, the most common methods used to track OTS include hormone tests, performance tests, psychological tests, and biochemical and immune markers (Meeusen et al., 2013). However, their practical application in the sports medicine field remains limited due to high costs, difficulty of implementation, lack of compliance from coaches and players, complexity, and the need for invasive measurement procedures.

Among noninvasive methods, heart rate variability (HRV) has emerged as a widespread investigational and clinical tool for indirectly evaluating autonomic modulation and neural adaptation to physical exercise, with potential applications in OTS screening (Billman, 2011; Khandoker et al., 2008; Santos‐Hiss et al., 2011). HRV corresponds to the study of the oscillations in the R‐R intervals or beat‐to‐beat variations of the electrocardiogram to explore the cardiac autonomic modulation. HRV can be assessed using linear methods, such as time or frequency domain techniques, which measure the balance between sympathetic and parasympathetic activity. Alternatively, nonlinear methods such as the Poincaré method, entropy, and detrended fluctuation analysis can also be employed. These nonlinear methods consider the complex interactions of biological systems on the heart (Khandoker et al., 2008; Shaffer & Ginsberg, 2017).

As a result, HRV has been recognized as a sensitive biomarker of both physiological and psychological systems, reflecting the ANS's ability to adapt to stressors, indicating good health, and being linked to executive function and sport performance (Gilgen‐Ammann et al., 2019; Jiménez Morgan & Molina Mora, 2017). It may be used to assess the stress of acute exercise, to modify the ANS because of exercise training, and to identify overtraining or overreaching (Hernando et al., 2018). It has also been reported that HRV may represent the training‐induced level of stress and recovery (Morales et al., 2014). Furthermore, a decrease in HRV is detrimental as it indicates inadequate adaptation of the ANS and has been linked to tiredness, stress, and overtraining (Kajaia et al., 2017). Therefore, despite the scientific interest in HRV monitoring, there is still a lack of understanding regarding HRV indices, their potential interpretation, and translational approaches in the clinical setting. Previous studies have demonstrated limited utilization, implementation, and different HRV usefulness among practitioners at different soccer clubs (Buchheit, 2014; Catai et al., 2019). A lack of consensus in HRV‐based assessment could be explained by unstandardized protocol measurements and frequency.

Therefore, the purpose of this study was to conduct a systematic review to establish whether there is an association between HRV indices and symptoms of overtraining in soccer athletes. According to our knowledge, this is the first systematic review on this topic, and the findings of this systematic review could be useful for sports professionals to track and prevent OTS through a feasible and noninvasive tool, as well as to elucidate the most used HRV indices in these players. Furthermore, the critical appraisal of the studies included in this review will help future research improve its methodological and evidence quality, addressing the existing limitations in the field. In addition, it represents a first step toward reaching a consensus and promoting standardization in this area of research.

## 2 METHODS

The Preferred Reporting Items for Systematic Reviews and Meta‐Analyses (PRISMA) guidelines (Supplementary File S1) were followed to conduct and report this study. Moreover, the systematic review protocol was registered with the International Prospective Register of Systematic Reviews (PROSPERO) under the following registration number: CRD42024513576. An electronic application for systematic review named Rayyan was used to systematize the screening (available from https://www.rayyan.ai).

### 2.1 Search strategy

A literature search was conducted from inception to February 5, 2024, on the following electronic databases: Web of Science, PubMed (via the National Library of Medicine), EMBASE, CINAHL, and SCOPUS (Elsevier). The following mesh terms were used and combined between them to perform the search strategy as follows: (Athletes OR “Athletes” [Mesh] OR “Sports” [Mesh]) AND (HRV OR HRV parameters OR HRV exercise training OR HRV biofeedback training OR Vagus nerve stimulation OR Autonomic Nervous System OR ANS OR Heart rate variab* OR Cardiac vagal acti* OR “Vagus Nerve Stimulation” [Mesh] OR “Autonomic Nervous System” [Mesh] OR “Vagus Nerve” [Mesh] OR “Sympathetic Nervous System” [Mesh]) AND (Overtrain* OR Overreach* OR “Overtraining Syndrome” [Mesh]). Additionally, the search strategy was limited to humans and English peer‐reviewed. In addition, randomized controlled trials, controlled clinical trials, longitudinal studies, prospective studies, cross‐sectional studies, and retrospective studies were included in this review; other study designs were excluded. A total of 2041 articles resulting from the search strategy were reviewed by two independent reviewers (C.L. and T.T.). Initially, articles were screened based on their title and abstract, following our eligibility criteria. Subsequently, the remaining articles that showed potential to meet the eligibility criteria were screened in full text. In the event of any discrepancies among these reviewers, a third independent reviewer (A.L.) was sought for consultation.

### 2.2 Eligibility criteria

The eligibility criteria were defined using the Sample (S)–Phenomenon of Interest (Pi)–Design (D)–Evaluation (E)–Research Type (R) (SPIDER) format. This systematic review focuses on the relationship between HRV parameters (Pi) and overtraining in soccer players (S). A qualitative research synthesis approach (R) is used to identify potential overtraining indices (E) derived from HRV analysis of linear and nonlinear methods. The eligible sample consisted of adult soccer players (>18 years old) who suffered from physical or psychological symptoms linked to overtraining. The term “athlete” as standardized. Athletes include individuals who engage in physical activity, ranging from “exercisers” to “elite athletes.” Four subcategories are established: “elite athlete,” “competitive athlete,” “recreational athlete,” and “exerciser” based on the following criteria: intent to compete, volume of exercise (hours per week), and level of competition (McKinney et al., 2019). This systematic review encompasses all stages that an overtrained athlete may encounter. The different phases are categorized as “functional overreaching” (FOR), “non‐functional overreaching” (NFOR), and “overtraining syndrome” (OTS) (Meeusen et al., 2013). Moreover, only English‐written studies and reporting statistical analysis were included.

### 2.3 Heart rate variability indexes

Table 1 lists the HRV indices used in the included studies, their acronyms, units of measurement, and interpretations (Shaffer & Ginsberg, 2017).

**TABLE 1:** HRV indices used in studies included in the systematic review.

HRV indices	Measure (units)	Description
Linear indices		
Time domain	SDNN (in ms)	Standard deviation of NN intervals (between NORMAL beats)
	SDRR (in ms)	Standard deviation of RR intervals (between NORMAL or ABNORMAL beats)
	RMSSD (in ms)	Root mean square of successive RR intervals differences
Frequency domain	VLF band (in ms^2^)	Absolute power of the very‐low‐frequency band. Power of LF values between 0.0033 and 0.04 Hz
	LF power (in ms^2^ normal units or %)	Power of the low‐frequency band (absolute or relative) LF values between 0.04 Hz and 0.15 Hz
	HF power (in ms^2^ normal units or %)	Power of the high‐frequency band (absolute or relative) HF values between 0.15 and 0.4 Hz
	LF/HF	LF‐HF power ratio
	TP	Variance of all R‐R intervals. Values ≤0.4 Hz
Nonlinear indices	Poincaré plot analysis	Scatter plot of every R‐R Interval against the prior interval
	S	Derivative functions of an ellipse which fits all the plotted points of the Poincaré Graph, and which represents total HRV
	SD1	
	SD2	
	SD1/SD2	SD1‐SD2 ratio which measures the unpredictability of the RR time series

Time‐domain method utilizes HRV indices to analyze the numerical analysis between successive RR intervals (RRi). This includes the standard deviation of NN intervals (SDNN), which represents the combined influence of sympathetic and parasympathetic modulation, and RMSSD, which specifically indicates cardiac parasympathetic modulation. SDNN shows a positive correlation with ultra‐low frequency (ULF), very low frequency (VLF), low frequency (LF), and total power (TP). Higher SDNN values typically indicate a healthier autonomic function characterized by balanced activity of the sympathetic and parasympathetic nervous systems. The RMSSD, the gold standard for assessing vagal activity, is closely related to HF and yields results similar to SD1.

Frequency‐domain measurements examine the allocation of absolute or relative power across various frequency ranges throughout a specific time frame. They can offer comprehensive data on the contributions of various physiological processes to HRV and distinguish the impacts of different branches of the autonomic nervous system. VLF represents the long‐term regulatory mechanisms (e.g., the renin‐angiotensin system) and provides insights into the body's longer‐term stress responses and regulatory mechanisms. LF represents the activity of the sympathetic nervous system and the regulation of blood pressure, associated with the activation of resting baroreceptors. However, LF power is considered a controversial index of sympathetic modulation, as the outcomes are also influenced by parasympathetic modulation (Appel et al., 1989). High‐frequency (HF) power represents parasympathetic activity, while the LF/HF ratio is a measure of the balance between sympathetic and parasympathetic cardiac modulation. Therefore, an increase in HF power at rest is generally associated with healthier autonomic function. Conversely, an elevated resting LF component, often considered to reflect a mix of sympathetic and parasympathetic influences, along with an increased LF/HF ratio, is sometimes interpreted as a marker of sympathetic predominance or autonomic imbalance. However, it is important to note that the interpretation of LF remains controversial in the literature, with many researchers considering it an index of mixed autonomic origin rather than a pure marker of sympathetic activity (Shaffer & Ginsberg, 2017). This imbalance is linked to poorer cardiovascular outcomes and increased cardiovascular risk. A higher LF/HF ratio indicates greater sympathetic dominance, while a lower ratio indicates greater parasympathetic dominance (Shaffer & Ginsberg, 2017). Finally, the TP index is related to all components of HRV across all frequencies and is a marker of variability of the NN intervals during the recording period (Shaffer & Ginsberg, 2017).

Nonlinear indexes analyze complex and constantly changing aspects of RRi fluctuations that are not considered in linear analysis. They offer data on the disordered and self‐repeating patterns and connections of cardiac variability. Poincaré Plot Analysis visualizes hidden time series patterns and is insensitive to R‐R trends. The size of the ellipse (S) covering all graph points relates to baroreflex sensitivity, LF, HF, and RMSSD. The ellipse's width (height) and length are SD1 and SD2. SD1/SD2 measures SNS‐activated autonomic balance. Regarding nonlinear indices, higher values in measures such as the Poincaré plot SD1, sample entropy (SampEn), and detrended fluctuation analysis (DFA α1) tend to be associated with greater complexity and adaptability of autonomic regulation, both indicative of healthier autonomic function. On the other hand, SD2 reflects long‐term variability and the combined influence of both autonomic branches. Additionally, the SD1/SD2 ratio serves as a marker of the balance between short‐ and long‐term variability, with reduced SD2 or SD1/SD2 ratio alterations often reported in cardiovascular conditions and considered a marker of poor autonomic regulation (Acharya et al., 2006; Voss et al., 2008, 2015).

### 2.4 Data extraction

Data were independently extracted by two reviewers (C.L. and T.T.) using standardized forms to ensure accuracy and minimize bias. Extracted information included measures of variability (e.g., mean and standard deviation), as well as correlation indices related to the association between HRV and OTS. All data were organized into structured tables to support synthesis and comparison. These included: (1) characteristics of studies/participants included in the systematic review and (2) correlations between HRV and OTS, presenting reported statistical associations.

### 2.5 Risk of bias

The methodological quality of each study was evaluated using the Joanna Briggs Institute (JBI) checklist (Moola et al., 2024), since most of our studies had a cross‐sectional design. The checklist comprises eight consecutive items, each with four options for box‐ticking: “yes” if the criterion was present, and “no,” “unclear,” or “not applicable” if the criterion was absent. The presence of a criterion is assigned a value of one point, whereas its absence is regarded as zero. The studies were classified into three levels according to their final score: low methodological quality (<5), fair methodological quality (5 to 6), or high methodological quality (≥7). Two independent reviewers (C.L. and T.T.) conducted the task of evaluating the risk of bias. In the event of any disagreement, a third researcher (A.L.) was consulted to achieve consensus.

## 3 RESULTS

### 3.1 Study selection

**FIGURE 1:**
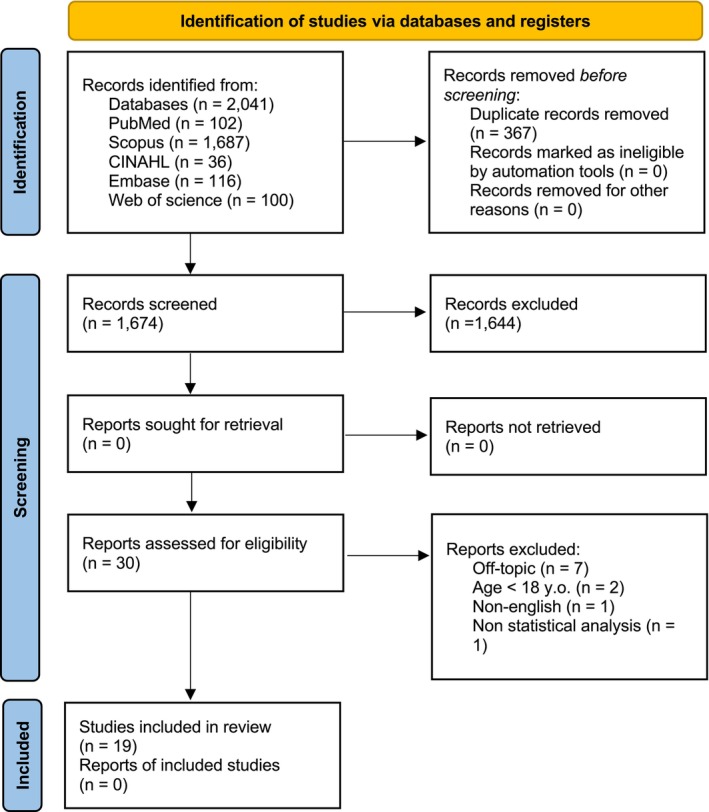
PRISMA flow diagram.

The electronic search revealed a total of 2041 articles. After removing duplicates, 1674 articles remained. Following the initial screening process, in which eligibility criteria were applied to the title and abstract only, 1644 articles were determined ineligible and excluded. As a result, a total of 30 publications were analyzed in full text during the subsequent phase, and 19 articles were included. The primary reasons for full text exclusion were off‐topic studies, the participation of individuals under the age of 18, and the absence of English language or statistical analysis. The PRISMA flowchart for study selection is shown in Figure 1.

### 3.2 Study characteristics

The characteristics of the participants and groups are described in Table 2.

**TABLE 2:** Characteristics of studies included in the systematic review.

First author, year, country	Study design	Period of observation	Population	Sample size	Gender (M/F)	Age mean ± SD (year)	Associations
Botelho, 2022, Brazil	Longitudinal study	Preseason (7 weeks)	Female elite soccer players	*n* = 24	F (0/24)	25.3 ± 3.81	HRV + psychophysiological changes
Costa, 2019, Portugal	Longitudinal study	In‐season (9 days of international tournament)	Female elite outfield soccer players	*n* = 20	F (0/20)	25.2 ± 3.1	Nocturnal HRV + sleeping patterns + competition
Costa, 2021, Portugal	Longitudinal study	In‐season (2 weeks)	Female high‐level outfield soccer players	*n* = 34	F (0/34)	20.6 ± 2.3	Sleep habits + nocturnal HRV + training and match load
Esco, 2016, USA	Longitudinal study	Off‐season (11 weeks)	Female soccer players	*n* = 9	F (0/9)	21.78 ± 2.04	HRV + ${\mathrm{VO}}_{2\mathrm{MAX}}$
Fields, 2021, USA	Longitudinal study	Preseason (2 weeks)	Male soccer players	*n* = 20	M (20/0)	20.3 ± 0.9	HRV + other internal and external load measures
Figueiredo, 2019, Brazil	Single‐group longitudinal study	Preseason (4 weeks)	Male soccer players	*n* = 16	M (16/0)	18.7 ± 0.6	HRV + overload
Flatt, 2016, USA	Single‐group longitudinal study	Off‐season (5 weeks)	Female soccer players	*n* = 12	F (0/12)	22 ± 2.3	Smartphone‐derived HRV + training load
Flatt, 2017, USA	Single‐group longitudinal study	Preseason (3 weeks)		*n* = 8	F (0/8)	20.2 ± 1.8	Smartphone‐derived HRV + training load
Flatt, 2017, USA	Longitudinal study	Off‐season (2 weeks)	Female soccer players	*n* = 10	F (0/10)	21.6 ± 2	Smartphone‐derived HRV + training load
Flatt, 2015, USA	Longitudinal study	Off‐season (3 weeks)	Female soccer players	*n* = 9	F (0/9)	22 ± 1.9	Smartphone‐derived HRV + training load
Marcelo de Queiroz Miranda, 2019, Brazil	Longitudinal study	Before and after a period of field soccer competition	Male professional soccer players	*n* = 17	M (17/0)	24 ± 3	HRV + competition
Morales, 2019, Spain	Longitudinal study	In‐season (Last mesocycle of competitive period)	Female professional soccer players	*n* = 16	F (0/16)	23.25 ± 5.07	HRV + training load + psychophysiological changes
Rabbani, 2019, Iran	Longitudinal study	In‐season (3 weeks) (On match day and the 4 following days)	Outfield male soccer players	*n* = 9	M (9/0)	25.2 ± 4.3	HRV + recovery + Hooper Index
Ravé, 2020, France	Longitudinal study	In‐season (12 days)	Male soccer players	*n* = 14	M (14/0)	27.9 ± 4.3	HRV + perceived physical fitness
Santos‐García, 2022, Spain	Longitudinal study	In‐season (3 micro cycles of competition)	Female soccer players	*n* = 8	F (0/8)	23.8 ± 4.5	Nocturnal HRV + TL + recovery
Sekiguchi, 2021, USA	Longitudinal study	In‐season (14 weeks)	Collegiate male soccer players	*n* = 23	M (23/0)	21 ± 1	HRV + ACWR
Thorpe, 2015, UK	Longitudinal study	In‐season (17 days)	Outfield male soccer players	*n* = 10	M (10/0)	19.1 ± 0.6	HRV + training load + fatigue
Thorpe, 2016, UK	Longitudinal study	In‐season (3 weeks)	Male soccer players	*n* = 29	M (29/0)	27 ± 5.1	HRV + training load + wellness status
Thorpe, 2017, UK	Longitudinal study	In‐season (17 days)	Outfield male soccer players	*n* = 10	M (10/0)	19.1 ± 0.6	HRV + training load + fatigue

Ten of the 19 studies were carried out in season, ranging from 9 days to 14 weeks (Costa et al., 2019, 2021; Morales et al., 2019; Rabbani et al., 2019; Ravé et al., 2020; Santos‐García et al., 2022; Sekiguchi et al., 2021; Thorpe et al., 2015, 2016, 2017). Four were conducted preseason, lasting between 2 and 7 weeks (Botelho et al., 2022; Fields et al., 2021; Figueiredo et al., 2019; Flatt, Esco, Nakamura, & Plews, 2017), and four off‐season, extending between 2 and 11 weeks (Esco et al., 2016; Flatt & Esco, 2015, 2016; Flatt, Esco, Nakamura, & Plews, 2017). Another study was carried out before and after a period of three mesocycles of competition lasting 7 weeks (Marcelo de Queiroz Miranda et al., 2019). The total sample size of the studies was 298 individuals (ranging from 8 to 34), with 150 females and 148 males. All participants were above 18 years old, with an average age of 23.2 years old.

Seven studies were conducted in the United States, including five on female soccer players, one during the preseason (Flatt, Esco, & Nakamura, 2017) and four during the off‐season (Esco et al., 2016; Flatt & Esco, 2015, 2016; Flatt, Esco, Nakamura, & Plews, 2017). Three of these five studies did not report their design (Esco et al., 2016; Flatt & Esco, 2015; Flatt, Esco, Nakamura, & Plews, 2017), while the other two were a single‐group observational study (Flatt, Esco, Nakamura, & Plews, 2017) and a single‐group correlation study (Flatt & Esco, 2016). The last two were a longitudinal study on male soccer players during the preseason (Fields et al., 2021) and an observational study on collegiate male soccer players during the in‐season (Sekiguchi et al., 2021). Four of the seven studies analyzed smartphone‐derived HRV associated with training load (Flatt & Esco, 2015, 2016; Flatt, Esco, & Nakamura, 2017; Flatt, Esco, Nakamura, & Plews, 2017). The last three studied HRV associated with ACWR (acute chronic workload ratio) (Sekiguchi et al., 2021), ${\mathrm{VO}}_{2\mathrm{MAX}}$ (maximal oxygen consumption) (Esco et al., 2016), and other internal and external load measures (Fields et al., 2021). Three studies were carried out in Brazil, including two observational studies during the preseason, one on female elite soccer players (Botelho et al., 2022) and the other on male soccer players (Figueiredo et al., 2019). The authors of these three studies investigated HRV associated with competition (Marcelo de Queiroz Miranda et al., 2019), overload (Figueiredo et al., 2019), and psychophysiological changes (Botelho et al., 2022). Three studies were conducted in the United Kingdom, all during in‐season on male soccer players (Thorpe et al., 2015, 2016, 2017). Two of them were conducted more specifically on outfield players (Thorpe et al., 2015, 2016). The studies focused on HRV associated with training load, fatigue (Thorpe et al., 2015, 2017), and wellness status (Thorpe et al., 2016). The design of these three studies was not reported. Two observational studies were carried out in Portugal, during in‐season on outfield female elite soccer players (Costa et al., 2019, 2021). The authors analyzed nocturnal HRV and sleeping patterns associated with competition (Costa et al., 2019), match, and training load (Costa et al., 2021). Two studies were carried out in Spain, both during in‐season. One studied nocturnal HRV in relation to training load and recovery in female soccer players. The other studied HRV, training load, and psychophysiological changes in professional female soccer players (Morales et al., 2019). Study designs were not reported. The last two studies took place during the in‐season. One was carried out in France and studied HRV associated with perceived physical fitness in male soccer players (Ravé et al., 2020). The other was carried out in Iran and studied HRV associated with recovery and the use of the Hooper index in outfield male soccer players (Rabbani et al., 2019). The designs of these two studies have not been reported.

### 3.3 Heart rate variability

The main HRV outcomes are listed in the penultimate column of Table 3.

**TABLE 3:** Data extraction table of studies included in the systematic review.

First author, year	OT assessment	HRV recording	Main HRV outcomes	Correlation—time domain and OT	Correlation—frequency domain and OT
Botelho, 2022	ITL Mood states Day and evening salivary testosterone and cortisol [C] Blood creatine kinase [CK]	POLAR, model S810	LF/HF: n.s SDNN: n.s RMSSD: n.s	N/A	Testosterone levels × LF/HF (+) Salivary cortisol levels × LF/HF (−)
Costa, 2019	s‐RPE Perceived ratings of wellbeing	Firstbeat Bodyguard2	LF: n.s HF: n.s RMSSD: n.s	Ø Correlation	N/A
Costa, 2021	s‐RPE TD Training and match exposure time (volume) HSR	Firstbeat Bodyguard2	LF: n.s HF: n.s LF/HF: n.s SDNN: n.s RMSSD: n.s SDRR: n.s	Ø Correlation	Ø Correlation
Esco, 2016	${\mathrm{VO}}_{2\mathrm{MAX}}$	Polar T‐31	RMSSD: n.s	lnRMSSD_M_ × ${\mathrm{VO}}_{2\mathrm{MAX}}$ (+)	N/A
Fields, 2021	s‐RPE	Polar H7	RMSSD: n.s	lnRMSSD × s‐RPE (−)	N/A
Figueiredo, 2019	TL ST Monotony Yo‐Yo IR1 Strain during the preseason	Suunto Memory Belt	OL lnRMSSD_mean_ values ↓ compared to BL OL lnRMSSD_cv_ ↑ compared to BL OL lnRMSSD_mean_ ↓ compared to TP OL lnRMSSD_cv_ values ↑ compared to TP	lnRMSSD_mean_ × TL for OL_1_ and OL_2_ (−) lnRMSSD_cv_ × TL for OL_1_(+), OL_2_(+) and TP (−) lnRMSSD_mean_ × Monotony for OL_1_, OL_2_ and TP (−) lnRMSSD_cv_ × Monotony for OL_1_(+), OL_2_ (−) and TP (+) lnRMSSD_mean_ × Strain for OL_1_, OL_2_ and TP (−) lnRMSSD_cv_ × Strain for OL1, OL2 and TP (+) lnRMSSD_mean_ × ∆%Yo‐Yo for OL_1_ and OL_2_ (+) lnRMSSD_cv_ × ∆%Yo‐Yo for OL_1_ and OL_2_ (−)	N/A
Flatt, 2016	ΔRHR_mean_ ΔRHR_cv_ Yo‐Yo IR1	Polar T‐31 noncoded	RMSSD: n.s	ΔLn rMSSDcv × ΔYo‐Yo (*r* = 20.74; *p* = 0.006)	N/A
Flatt, 2017	TTL DTL Fatigue	Polar T‐31 noncoded	lnRMSSD ↑ ES = 0.35 *p*‐values: N/R	lnRMSSD × TTL (*r* = −0.86) lnRMSSD × DTL (*r* = −0.85) lnRMSSD × fatigue (*r* = +0.56) lnRMSSD × soreness (*r* = +0.54) lnRMSSD × sleep (*r* = +0.34) *p*‐values: N/R	N/A
Flatt, 2017	TL Psychometric Data	Polar T‐31 noncoded	RMSSD: ↓ Friday compared to the previous days on the High Load Group ES = −0.64 ± 0.78 *p*‐values: N/R	lnRMSSDcv × fatigue (*r* = +0.55) lnRMSSDcv × fitness levels (*r* = −0.61 for VO_2max_; *r* = −0.65 for Yo‐Yo) *p*‐values: N/R	N/A
Flatt, 2015	Smartphone‐derived + training load	Polar T‐31 noncoded	3‐day supine CV ↑ compared to low load group (*p* = 0.003; ES = 0.86)	N/A	N/A
Miranda, 2019	Competition	Polar S810i	LF: ↓ (*p* < 0.05; ES = 1.86) HF: ↓ (*p* < 0.05; ES = 0.89) LF/HF: ↓ (*p* < 0.05; ES = 1.86) SDNN: ↓ (*p* < 0.05; ES = 1.86) RMSSD: n.s SDRR: ↓ (*p* < 0.05; ES = 1.86)	N/A	N/A
Morales, 2019	RESTQ‐sport test Cooper test Yo‐Yo IR1 Monotony	Polar RS810	LF: ↑ (*p* = 0.001) HF: ↓ (*p* = 0.001) LF/HF: ↑ (*p* = 0.001) RMSSD: n.s SDRR: n.s ES: N/R	ΔRMSSD × ΔYo‐Yo IR1 (+) ΔRMSSD × Cooper Test (+) (*r* = 0.78; *p* = 0.03) ΔRMSSD × Δgeneral stress (−) (*r* = −0.61; *p* = 0.01) ΔRMSSD × Δspecific stress (+) (*r* = 0.58; *p* = 0.01) ΔRMSSD × Δgeneral recovery (+) (*r* = 0.64; *p* = 0.003) ΔRMSSD × Δspecific recovery (+) (*r* = 0.50; *p* = 0.009)	ΔHF and Δspecific recovery (+) (*r* = 0.68; *p* = 0.007) ΔHF × Δgeneral stress (+) (*r* = 0.55; *p* = 0.02) ΔHF and Δspecific stress (+) (*r* = 0.61; *p* = 0.02) ΔLF × Δspecific Stress (+) (*r* = 0.38; *p* = 0.04) ΔLF/HF × Δspecific recovery (+) (*r* = 0.48; *p* = 0.01) ΔLF/HF × Δspecific stress (+) (*r* = 0.31; *p* = 0.04) ΔLF/HF × Δgeneral stress (+) (*r* = 0.55; *p* = 0.04)
Rabbani, 2019	Hooper Index TE SWC TE/SWC	Polar H7	RMSSD: n.s	Hooper Index × lnRMSSD (−) (*r* = −0.41)	N/A
Ravé, 2020	VAS	PolarTeamSystem2	N/A	RMSSD × VAS (+) (*r* > 0.4; *p* < 0.01) RMSSD × TP (+) (*r* = 0.861; *p* < 0.001) RMSSD × HF (+) (*r* = 0.938; *p* < 0.001)	TP × VAS (+) (*r* > 0.45; *p* < 0.01) LF × VAS (+) (*r* > 0.50; *p* < 0.001) LF_nu_ × VAS (+) (*r* = 0.390; *p* < 0.01) HF × VAS (+) (*r* = 0.442; *p* < 0.01) HF_nu_ × VAS (−) (*r* = −0.396; *p* < 0.01) LF/HF × VAS (+) (*r* = 0.391; *p* < 0.01)
Santos‐García, 2022	TL RHR HIR distance Psychometric Test	Firstbeat Bodyguard 2	RMSSD: ↑ in Post 1 (*p* < 0.01) and Post 2 (*p* < 0.05) compared to Match Day ↑ in Day 2 compared to Day 1 (*p* < 0.01 for 4 h and *p* < 0.05 for 5 min recording) ↓ in Day 3 compared to Day 2 (for 4 h and 5 min recording) ↑ in Day 3 compared to Day 1 (*p* < 0.05 for 4 h and 5 min recording) ↑ in Day 23 compared to Day 22 (*p* < 0.01 for 4 h recording) ↑ in Day 18 compared to Day 17 (*p* < 0.01 for 5 min recording) ES: N/R	RMSSD_4h_ × RHR_4h_ (+) (*r* = +0.93 and *p* < 0.001) RMSSD_5min_ × RHR_5min_ (+) (*r* = +0.85 and *p* = 0.01) RMSSD_4h_ × Psychometric Results (−) (*r* = −0.75 and *p* = 0.03) SDNN × RHR_5min_ (+) (*r* = −0.75; *p* = 0.03)	N/A
Sekiguchi, 2021	ACWR	Polar Team 2	RMSSD: ↑ in W12 compared to W1 (*p* = 0.026, ES = 0.87)	RMSSD × ACWR_ST_ (−) (*r* = −7.4 and *p* = 0.04)	N/A
Thorpe, 2015	THIR distance	Polar precision performance	RMSSD: ↓ (*p* = 0.05; ES = 12%)	lnRMSSD × THIR (−) (*r* = −0.24, *p* = 0.04)	N/A
Thorpe, 2016	THIR distance	Polar precision performance	RMSSD: n.s	Ø Correlation	N/A
Thorpe, 2017	RPE‐TL Psychometric Test	Polar precision performance	RMSSD: n.s	N/A	N/A

Abbreviations: [C], concentration; ACWR, acute chronic workload ratio; ACWR_ST, acute chronic workload ratio session time; DTL, day training load; HF, high frequency; HSR, high‐speed running; ITL, internal training load; ln, natural logarithm; n.s., not significant; N/A, not assessed; N/R, not reported; OL, week(s) of over load; RHRcv, resting heart rate (cv); RHRmean, resting heart rate (mean); RMSSD, root mean square of successive differences; s‐RPE, session‐rate perceived exertion; SS, stress score; ST, stress tolerance; SWC, smallest worthwhile change; TD, total distance; TE, typical error; THIR, total high‐intensity‐running; TP, tapering period; TP, total power; TRIMP, training impulse; TTL, total training load; Unclear Correlations, occur when the relationship between variables cannot be precisely determined due to complex data distributions or overlapping confidence intervals, making the true magnitude of the correlation uncertain; VLF, very low frequency; Yo‐Yo IR1, Yo‐Yo intermittent recovery test (level 1).

Among the selected articles, none reported nonlinear HRV indices. All articles present results of linear indices, such as frequency and time domain analysis, with nine studies demonstrating significant differences in HRV parameters postintervention (i.e., after training, match, and/or tapering period) (Sekiguchi et al., 2021; Thorpe et al., 2017). RMSSD is the most frequently measured HRV parameter, with 16 studies acquiring it. Six studies among them demonstrate significant differences in the RMSSD parameter, either in its absolute or logarithmic form. RMSSD significantly decreases on match days and increases again on subsequent days, indicating an acute reduction in cardiac parasympathetic modulation, which typically recovers within 2 days (Santos‐García et al., 2022). Regarding $\ln{\text{RMSSD}}_{\mathrm{cv}}$, that is, the natural logarithm of RMSSD, two studies report a significant increase in this parameter following intensive training (Figueiredo et al., 2019; Flatt & Esco, 2015), while another one shows a significant decrease in the days following a match (Thorpe et al., 2015). An increase in $\ln{\text{RMSSD}}_{\mathrm{cv}}$, may indicate improved autonomic adaptability and a healthy training response, while excessive variability suggests inadequate recovery or overtraining. On the other hand, a decrease postmatch may reflect reduced autonomic flexibility, fatigue, or prolonged recovery needs. For $\ln{\text{RMSSD}}_{\text{mean}}$, one of the studies selected reports a significant increase at the end of the preseason (Flatt, Esco, Nakamura, & Plews, 2017), suggests improved parasympathetic recovery and overall cardiovascular health, indicating a positive adaptation to training. Meanwhile, two other studies indicate a decrease in this index during intensive training (Figueiredo et al., 2019; Flatt, Esco, Nakamura, & Plews, 2017), which may signal autonomic suppression, potentially indicating overtraining, which could negatively affect long‐term health if sustained.

Considering frequency domain indexes, four analyzed LF, HF, and the LF/HF ratio parameters, but only two report significant results. One study shows a significant decrease in all three parameters after competitions among professional athletes (Marcelo de Queiroz Miranda et al., 2019), while another study reveals an increase in LF and the LF/HF ratio and a decrease in HF after training (Morales et al., 2019). These responses may indicate reduced parasympathetic activity and possible increased sympathetic dominance. This shift could be a sign of autonomic imbalance, potentially impairing recovery and overall cardiovascular health. Finally, only one study analyzes TP, but solely includes a correlational analysis (Ravé et al., 2020).

### 3.4 HRV time domain indexes and overtraining

The primary results of the correlation study are presented in Table 3. Among the included studies, 13 demonstrate correlations between linear HRV parameters and symptoms of overtraining, leading to the identification of 42 distinct significant associations. Thirteen of these correlations involved HRV frequency domain parameters, while the other 28 involved HRV time domain characteristics. One study revealed a significant inverse relationship between the total HRV and ${\text{ACWR}}_{\mathrm{ST}}$ (acute chronic workload ratio session time) (Sekiguchi et al., 2021), suggesting that higher training loads relative to chronic workload may lead to reduced HRV. This could indicate increased sympathetic dominance and decreased parasympathetic activity, potentially reflecting insufficient restoration. Out of the time domain indices examined, only one study demonstrates a significant correlation between SDNN and an indication of overtraining (Santos‐García et al., 2022). The study shows that during a competition phase, SDNN changes in the same direction as the resting heart rate after 5 min of patient stabilization.

RMSSD is significantly correlated with different symptoms of overtraining. Regarding the change in RMSSD before and after the intervention, three studies have found a positive association with Yo‐Yo IR1, the Cooper Test, general recovery, sport‐related stress, visual analog scale (VAS), and resting heart rate (RHR) (Morales et al., 2019; Ravé et al., 2020; Santos‐García et al., 2022). However, they have observed a negative association with general stress and psychometric data, suggesting that while improved RMSSD may reflect better physical performance and recovery, it could also indicate heightened psychological strain. This disparity highlights the complex interplay between physiological recovery and mental stress, suggesting that improvements in autonomic function may not always translate to overall well‐being, especially when mental fatigue or stress are not adequately addressed. Regarding $\ln{\text{RMSSD}}_{\text{mean}}$, several studies (Esco et al., 2016; Fields et al., 2021; Figueiredo et al., 2019; Flatt, Esco, & Nakamura, 2017; Rabbani et al., 2019; Thorpe et al., 2015) have found a positive association with variables such as ${\mathrm{VO}}_{2\mathrm{MAX}}$, fatigue, the Yo‐Yo IR1 during an overload period, soreness, and sleep. However, these studies have also observed a negative association between $\ln{\text{RMSSD}}_{\text{mean}}$ and variables such as s‐RPE, monotony (lack of TL variability), strain, training load (TL) during an overload period, the Hooper Index, and total high‐intensity‐running (THIR) distance. While improved $\ln{\text{RMSSD}}_{\text{mean}}$, may indicate better physical performance and recovery, the negative associations with certain training load parameters and perceived strain suggest that an increase in this measure could also reflect excessive training. This highlights the need for a balanced approach to training, as excessively high training loads may impair autonomic function and recovery despite positive physical outcomes.

In relation to $\ln{\text{RMSSD}}_{\mathrm{cv}}$, three studies established a positive correlation with TL during periods of overload, strain, and fatigue, while observing a negative correlation with TL during the tapering phase, the Yo‐Yo IR1, and fitness level (Figueiredo et al., 2019; Flatt & Esco, 2016; Flatt, Esco, Nakamura, & Plews, 2017), suggesting that excessive monotony or imbalanced training loads may impair recovery and performance. According to one of the latter, monotony is a unique situation where it is directly related to $\ln{\text{RMSSD}}_{\mathrm{cv}}$ in the first week of overload and the tapering phase but negatively related in the second week of overload (Figueiredo et al., 2019), which underscores the importance of managing both training intensity and variability to optimize recovery and prevent potential negative effects on autonomic function.

Finally, three studies reported nonsignificant correlations between OTS and time‐domain HRV indices, specifically RMSSD, SDRR, and SDNN parameters.

### 3.5 HRV frequency domain indexes and overtraining

Three studies demonstrated a notable association with frequency domain indices (Botelho et al., 2022; Morales et al., 2019; Ravé et al., 2020). LF shows a positive correlation with sport‐specific stress and with VAS (Morales et al., 2019; Ravé et al., 2020). On the other hand, HF is positively associated with general stress and sport‐related recovery (Morales et al., 2019) but is also inversely correlated with VAS (Ravé et al., 2020). Therefore, LF may indicate increased sympathetic activation due to sport‐related stress, while the positive association of HF with recovery suggests a beneficial parasympathetic response. However, the inverse correlation between HF and VAS implies that elevated HF may not always reflect optimal recovery, especially when coupled with stress or discomfort, highlighting the need for balance between sympathetic and parasympathetic activity for optimal performance.

Regarding frequency domain indexes, one study established a positive correlation between the LF/HF ratio and testosterone levels, as well as a negative correlation with salivary cortisol levels, which align with improved autonomic balance and physical recovery (Botelho et al., 2022). Moreover, a direct correlation is shown between this ratio, overall recovery, and stress connected to sports (Morales et al., 2019); and a link is established between LF/HF and VAS, which might also imply that variations in the LF/HF ratio could reflect psychological factors influencing perceived recovery (Ravé et al., 2020). This highlights the need for caution when using LF/HF as a sole indicator of recovery, as mental stress may contribute significantly to changes in this ratio. Finally, the two studies demonstrated a strong correlation between TP, sport recovery, and VAS (Morales et al., 2019; Ravé et al., 2020).

Although some associations have been reported, most included studies did not examine correlations between OTS markers and HRV frequency domain indexes. Moreover, only one study reported nonsignificant correlations, particularly for the LF, HF, and LF/HF ratio parameters.

### 3.6 Risk of bias

The average JBI checklist score of 19 studies is 6.3, indicating a fair methodology quality, as shown in Table 4. Eleven studies had high methodological quality, with a score of 7 or above. The rest of the studies had fair methodological quality (from 5 to 6), except for one study (Ravé et al., 2020), which had a low methodological quality (<5). Among these, the mode score was 7, observed in nine studies (47%) (Costa et al., 2019; Esco et al., 2016; Figueiredo et al., 2019; Morales et al., 2019; Santos‐García et al., 2022; Sekiguchi et al., 2021; Thorpe et al., 2015, 2016, 2017). All 19 studies presented coherent results in adequately describing the study sample and its features, the method of measurement of exposure (validity and reliability), the specific diagnosis or definition of patients included, the process and objectivity of the outcome measurement instrument, and the appropriateness of the statistical and analytical strategy. However, three questions on the JBI checklist revealed inconsistencies. Eight out of the 19 studies (42%) showed a lack of identification and addressing of confounding factors (Flatt & Esco, 2015; Flatt & Esco, 2016; Flatt, Esco, & Nakamura, 2017; Flatt, Esco, Nakamura, & Plews, 2017; Marcelo de Queiroz Miranda et al., 2019; Rabbani et al., 2019; Ravé et al., 2020). Finally, most of the articles (10 studies, 53%) did not provide any information about the eligibility criteria (Costa et al., 2019; Fields et al., 2021; Figueiredo et al., 2019; Flatt & Esco, 2015, 2016; Morales et al., 2019; Rabbani et al., 2019; Ravé et al., 2020; Santos‐García et al., 2022; Sekiguchi et al., 2021), and five studies (26%) had an unclear description of the criteria (Flatt, Esco, & Nakamura, 2017; Flatt, Esco, Nakamura, & Plews, 2017; Thorpe et al., 2015, 2016, 2017). Therefore, only four studies (21%) provided a description of their eligibility criteria (Botelho et al., 2022; Costa et al., 2021; Flatt & Esco, 2016; Marcelo de Queiroz Miranda et al., 2019).

**TABLE 4:** Methodological quality assessment according to the Joanna Briggs Institute (JBI) criteria.

Reference	Q1	Q2	Q3	Q4	Q5	Q6	Q7	Q8	Final score
Botelho et al. (2022)	Yes	Yes	Yes	Yes	Yes	Yes	Yes	Yes	8
Costa et al. (2019)	No	Yes	Yes	Yes	Yes	Yes	Yes	Yes	7
Costa et al. (2021)	Yes	Yes	Yes	Yes	Yes	Yes	Yes	Yes	8
Esco et al. (2016)	No	Yes	Yes	Yes	Yes	Yes	Yes	Yes	7
Fields et al. (2021)	No	Yes	Yes	Yes	No	No	Yes	Yes	5
Figueiredo et al. (2019)	No	Yes	Yes	Yes	Yes	Yes	Yes	Yes	7
Flatt and Esco (2016)	Yes	Yes	Yes	Yes	No	No	Yes	Yes	6
Flatt, Esco, and Nakamura (2017); Flatt, Esco, Nakamura, and Plews (2017)	NC	Yes	Yes	Yes	No	No	Yes	Yes	5
Flatt, Esco, and Nakamura (2017); Flatt, Esco, Nakamura, and Plews (2017)	NC	Yes	Yes	Yes	No	No	Yes	Yes	5
Flatt and Esco (2015)	No	Yes	Yes	Yes	No	No	Yes	Yes	5
Marcelo de Queiroz Miranda et al. (2019)	Yes	Yes	Yes	Yes	No	No	Yes	Yes	6
Morales et al. (2019)	No	Yes	Yes	Yes	Yes	Yes	Yes	Yes	7
Rabbani et al. (2019)	No	Yes	Yes	Yes	No	No	Yes	Yes	5
Ravé et al. (2020)	No	NC	Yes	Yes	No	No	Yes	Yes	4
Santos‐García et al. (2022)	No	Yes	Yes	Yes	Yes	Yes	Yes	Yes	7
Sekiguchi et al. (2021)	No	Yes	Yes	Yes	Yes	Yes	Yes	Yes	7
Thorpe et al. (2015)	NC	Yes	Yes	Yes	Yes	Yes	Yes	Yes	7
Thorpe et al. (2016)	NC	Yes	Yes	Yes	Yes	Yes	Yes	Yes	7
Thorpe et al. (2017)	NC	Yes	Yes	Yes	Yes	Yes	Yes	Yes	7

*Note*: The scoring was determined by assigning 1 point for a “yes” answer and 0 points for a “no,” “NC,” or “NA” answer to the following questions: (1) Were the criteria for inclusion in the sample clearly defined? (2) Were the study subjects and setting described in detail? (3) Was the exposure measured in a valid and reliable way? (4) Were objective, standard criteria used for the measurement of the condition? (5) Were confounding factors identified? (6) Were strategies to deal with confounding factors stated? (7) Were the outcomes measured in a valid and reliable way? (8) Was appropriate statistical analysis used? JBI checklist, methodological categories: low (<5); fair (5, 6); high (≥7).

Abbreviations: NA, not applicable; NC, not clear.

## 4 DISCUSSION

This systematic review aimed to determine whether there is a correlation between HRV indices and symptoms of overtraining in soccer athletes. To the authors' knowledge, this is the first systematic review conducted to analyze the potential correlations between HRV parameters and symptoms of overtraining in soccer athletes. Among the 19 included studies, the findings revealed a correlation between HRV parameters and physical performance, demonstrating that HRV can be a marker of OTS. Positive associations were observed between RMSSD and several clinical and field tests, such as ${\mathrm{VO}}_{2\mathrm{MAX}}$, Yo‐Yo IR1, and Cooper tests (Esco et al., 2016; Figueiredo et al., 2019; Morales et al., 2019). Several HRV parameters, including SDNN, RMSSD, HF, TP, LF, and LH/HF ratio, have shown a positive correlation with fatigue and recovery factors such as soreness, fatigue, RHR, general/specific recovery, sleep, and VAS (Flatt, Esco, & Nakamura, 2017; Flatt, Esco, Nakamura, & Plews, 2017; Morales et al., 2019; Ravé et al., 2020; Santos‐García et al., 2022). Additionally, hormonal markers showed correlations with HRV parameters, specifically the LF/HF ratio (Botelho et al., 2022). In addition, when considering psychological factors, the frequency domains LF, HF, and the LF/HF ratio all showed a positive correlation with general or specific stress (Morales et al., 2019). On the other hand, RMSSD also showed positive correlations with sleep and specific stress (Flatt, Esco, & Nakamura, 2017; Morales et al., 2019), but negative correlations with factors such as hooper index, general stress, and psychometric data (Morales et al., 2019; Rabbani et al., 2019; Santos‐García et al., 2022).

### 4.1 HRV and physical performance

It is known that higher resting cardiac parasympathetic modulation is essential for the proper functioning of the recovery processes in athletes (Buchheit et al., 2012). This increased vagal activity is associated with improved circulatory function and enhanced venous return, which supports more effective removal of metabolic products from tissues. It also reduces systemic inflammation and oxidative stress, which are involved in waste product accumulation and clearance (Pavlov & Tracey, 2012). Additionally, increased vagal activity has been associated with improved kidney function, which facilitates the secretion of metabolic wastes such as urea and creatinine (Pavlov & Tracey, 2012). On the other hand, higher levels of cardiac sympathetic activity have been related to higher stress and longer recovery periods. OTS is characterized by an imbalance between training and recovery, leading to increased adrenaline and noradrenaline levels, which suggest sympathetic activation. Consequently, the athlete is likely to develop fatigue, reduced performance, and vulnerability to injuries (Pavlov & Tracey, 2012). It was also found that sports professionals can identify the signs of overtraining based on cardiac sympathetic activity indices and modify the training loads to prevent the deterioration in athletic performance (Miguel et al., 2021).

Studies have shown that the link between higher baseline RMSSD is characterized by increased aerobic fitness and performance based on ${\mathrm{VO}}_{2\mathrm{MAX}}$ (Malagù et al., 2021). It is known that exercise capacity is mainly determined by coordinated interactions among the ventilatory, cardiovascular, and muscle‐skeletal systems. These interactions guarantee efficient oxygen uptake and transportation, which are essential for meeting the body's metabolic needs during exercise (Petek et al., 2021). The standard approach for assessing exercise capacity involves measuring ${\mathrm{VO}}_{2\mathrm{MAX}}$, which is determined by cardiac output and the difference between arterial and mixed venous oxygen concentrations (Petek et al., 2021). Therefore, variations in ${\mathrm{VO}}_{2}$ levels can be attributed to changes in the autonomic nervous system, since cardiac output is influenced in part by central command, which regulates locomotor, cardiovascular, and ventilatory responses during exercise (Patel & Zwibel, 2024). During training, an elevation in heart rate reserve when at rest can be related to either an augmentation in vagal activity or a reduction in sympathetic activity. This enhances the heart's cardiac output, leading to a condition of optimal balance, which allows for reaching for the highest levels of ${\mathrm{VO}}_{2\mathrm{MAX}}$ (Michael et al., 2017).

Cardiac autonomic regulation also plays a role in the perceived exertion during exercise. A reduced HRV is associated with higher sympathetic and lower parasympathetic activity, reflecting a state of autonomic imbalance. This imbalance is the marker of an incomplete recovery, which explains the increased rating of perceived exertion during exercise. This can reduce an athlete's preparedness and ability to maintain high‐intensity activities (Rave et al., 2018). On the other hand, high HRV is associated with a reduced rate of perceived exertion, which allows athletes to sustain high performances for a longer time (Ravé et al., 2020).

### 4.2 HRV and psychological aspects

Several outcome measures have been used to assess the link between HRV and the psychological aspect of athletes, such as the Hooper index, the RESTQ‐sport questionnaire, and psychometric tests assessing fatigue, muscle soreness, sleep quality, mood, and stress. Stress can be divided into two categories: anticipatory and acute stress in real‐life situations or chronic stress caused by depression, rumination, emotional exhaustion, and burnout. Lower physiological and emotional arousal regulation is associated with an increased response to stress, resulting in a lower resting HRV (Da Estrela et al., 2021). Competitive sport, which provokes anxiety due to the adaptation of the training load and the anticipation of competition phases, is part of anticipatory stress in a real situation (Immanuel et al., 2023). RMSSD has been reported as the most valid parameter for assessing emotional state in the precompetitive period. When the athlete experiences stress, the sympathetic nervous system becomes more active, leading to an increased heart rate and reduced HRV, as reflected by a lower RMSSD (Cervantes et al., 2009).

Considering sleep quality aspects, the LF/HF ratio has been often used to assess changes in autonomic function during sleep. Generally, an increase in sympathetic activity leads to an increased LF/HF ratio synonymous with poorer sleep quality (Stein & Pu, 2012). While the HF index is a potential index of vulnerability to sleep disturbances (Da Estrela et al., 2021). A greater activation of the sympathetic nervous system, resulting in an increased LF index, is associated with poor sleep quality. This phenomenon can disrupt the ability of the ANS to inhibit sympathetic dominance by reducing the vagal tone and HRV (Oliver et al., 2020).

### 4.3 Limitations and practical application

One significant limitation in the current literature is the lack of consistency in the HRV indexes and overtraining variables used across studies, which increases the heterogeneity and restricts the ability to draw definitive conclusions or conduct a meta‐analysis. This methodological variability contributes to the conflicting results observed in the literature, making it challenging to establish clear, generalizable patterns. Furthermore, the use of different population groups (e.g., elite athletes vs. collegiate athletes and male vs. female) and training protocols further limits the ability to compare findings. For this reason, a formal assessment of publication bias was not conducted in this review, as such evaluations typically require statistical methods applicable to meta‐analyses, such as Egger's Regression Test. Many of the studies reviewed report on different HRV parameters and use varying methods for assessing overtraining symptoms, such as subjective measures of fatigue, soreness, and training load.

However, it is important to note that despite this heterogeneity, certain HRV measures, such as RMSSD, consistently appeared across multiple included studies, and important correlations between this index and overtraining markers were found in several of them. This consistency suggests that RMSSD could be recommended for monitoring overtraining in athletes. Nonetheless, without standardized protocols and more consistent methodologies, drawing definitive conclusions or performing robust meta‐analyses remains a challenge.

## 5 CONCLUSION

This systematic review revealed several significant correlations between HRV parameters and markers of OTS in soccer players. The study showed potential associations between HRV parameters, particularly RMSSD, and physical performance indicators, including clinical and field tests and training load. A larger number of linear HRV parameters, such as SDNN, RMSSD, HF, TP, LF, and LH/HF ratio, demonstrated a correlation with fatigue and recovery factors. Psychological aspects also showed correlations with HRV parameters, including the LF/HF ratio, LF, HF, and RMSSD. However, the variability in HRV measurement methods and the absence of standardized criteria for diagnosing OTS limit the ability to establish HRV as a definitive marker of OTS. The differing methodologies across studies contribute to inconsistent findings, highlighting the need for standardized HRV assessment protocols and more rigorous studies to enable future meta‐analyses. Moreover, future research should further explore the applicability of nonlinear HRV indices in the context of OTS, as they can provide additional insights into the complexity and variability of autonomic regulation.

Furthermore, future studies should address confounding factors that might affect HRV, such as the menstrual cycle, smoking, alcohol, and caffeine (De Zambotti et al., 2013). Additionally, gender may influence the analysis and interpretation of HRV parameters, with women having a greater parasympathetic influence on cardiac regulation as opposed to men, who display sympathetic dominance (Schiweck et al., 2022). Moreover, some included studies were not carried out during the same period of the season, which can impact the results (Koenig & Thayer, 2016).

## AUTHOR CONTRIBUTIONS

RMdA, BC, CL, AL, and TT conceived and designed the study; RMdA, CL, AL, TT, and BC drafted the manuscript; RMdA, CL, AL, TT, and BC edited and revised the manuscript; and all the authors approved the final version of the manuscript.

## FUNDING INFORMATION

No sources of funding were used for this study.

## CONFLICT OF INTEREST STATEMENT

The authors declare no conflicts of interest.

## ETHICS APPROVAL

Not applicable.

## Supporting information


Data S1.

## Data Availability

Data are available from the authors on request. The PRISMA checklist can be found in Supplementary File S1.

## References

[phy270357-bib-0001] Acharya, U. R. , Joseph, K. P. , Kannathal, N. , Lim, C. M. , & Suri, J. S. (2006). Heart rate variability: A review. Medical & Biological Engineering & Computing, 44(12), 1031–1051. 10.1007/S11517-006-0119-0 /METRICS. 17111118

[phy270357-bib-0002] Appel, M. L. , Berger, R. D. , Saul, J. P. , Smith, J. M. , & Cohen, R. J. (1989). Beat to beat variability in cardiovascular variables: Noise or music? Journal of the American College of Cardiology, 14(5), 1139–1148. 10.1016/0735-1097(89)90408-7 2681319

[phy270357-bib-0003] Armstrong, L. E. , Bergeron, M. F. , Lee, E. C. , Mershon, J. E. , & Armstrong, E. M. (2021). Overtraining syndrome as a complex systems phenomenon. Frontiers in Network Physiology, 1, 794392. 10.3389/FNETP.2021.794392 36925581 PMC10013019

[phy270357-bib-0004] Billman, G. E. (2011). Heart rate variability—A historical perspective. Frontiers in Physiology, 2, 86. 10.3389/FPHYS.2011.00086 22144961 PMC3225923

[phy270357-bib-0005] Botelho, R. , Abad, C. C. C. , Spadari, R. C. , Winckler, C. , Garcia, M. C. , & Guerra, R. L. F. (2022). Psychophysiological stress markers during preseason among elite female soccer players. Journal of Strength and Conditioning Research, 36(6), 1648–1654. 10.1519/JSC.0000000000003702 35622110

[phy270357-bib-0006] Buchheit, M. (2014). Monitoring training status with HR measures: Do all roads lead to Rome? Frontiers in Physiology, 5, 73. 10.3389/FPHYS.2014.00073 24578692 PMC3936188

[phy270357-bib-0007] Buchheit, M. , Simpson, M. B. , Al Haddad, H. , Bourdon, P. C. , & Mendez‐Villanueva, A. (2012). Monitoring changes in physical performance with heart rate measures in young soccer players. European Journal of Applied Physiology, 112(2), 711–723. 10.1007/S00421-011-2014-0 21656232

[phy270357-bib-0008] Catai, A. M. , Pastre, C. M. , de Godoy, M. F. , da Silva, E. , Takahashi, A. C. d. M. , & Vanderlei, L. C. M. (2019). Heart rate variability: Are you using it properly? Standardisation checklist of procedures. Brazilian Journal of Physical Therapy, 24(2), 91. 10.1016/J.BJPT.2019.02.006 30852243 PMC7082649

[phy270357-bib-0009] Cervantes, J. , Rodas, G. , & Capdevila Ortís, L. (2009). Heart‐rate variability and precompetitive anxiety in swimmers. Psicothema, 21(4), 531–536. https://www.researchgate.net/publication/38040305_Heart‐rate_variability_and_precompetitive_anxiety_in_swimmers 19861094

[phy270357-bib-0010] Costa, J. , Figueiredo, P. , Nakamura, F. , Rago, V. , Rebelo, A. , & Brito, J. (2019). Intra‐individual variability of sleep and nocturnal cardiac autonomic activity in elite female soccer players during an international tournament. PLoS One, 14(9), e0218635. 10.1371/journal.pone.0218635 31527865 PMC6748428

[phy270357-bib-0011] Costa, J. A. , Figueiredo, P. , Nakamura, F. Y. , Rebelo, A. , & Brito, J. (2021). Monitoring individual sleep and nocturnal heart rate variability indices: The impact of training and match schedule and load in high‐level female soccer players. Frontiers in Physiology, 12, 678462. 10.3389/fphys.2021.678462 33981255 PMC8110215

[phy270357-bib-0012] Da Estrela, C. , McGrath, J. , Booij, L. , & Gouin, J. P. (2021). Heart rate variability, sleep quality, and depression in the context of chronic stress. Annals of Behavioral Medicine, 55(2), 155–164. 10.1093/ABM/KAAA039 32525208 PMC7962885

[phy270357-bib-0013] De Zambotti, M. , Nicholas, C. L. , Colrain, I. M. , Trinder, J. A. , & Baker, F. C. (2013). Autonomic regulation across phases of the menstrual cycle and sleep stages in women with premenstrual syndrome and healthy controls. Psychoneuroendocrinology, 38(11), 2618–2627. 10.1016/J.PSYNEUEN.2013.06.005 23850226 PMC3812396

[phy270357-bib-0014] Dellal, A. , Lago‐Peñas, C. , Rey, E. , Chamari, K. , & Orhant, E. (2015). The effects of a congested fixture period on physical performance, technical activity and injury rate during matches in a professional soccer team. British Journal of Sports Medicine, 49(6), 390–394. 10.1136/BJSPORTS-2012-091290 23422422

[phy270357-bib-0015] Esco, M. R. , Flatt, A. A. , & Nakamura, F. Y. (2016). Initial weekly HRV response is related to the prospective change in VO_2max_ in female soccer players. International Journal of Sports Medicine, 37(6), 436–441. 10.1055/s-0035-1569342 27042998

[phy270357-bib-0016] Fields, J. B. , Merigan, J. M. , Gallo, S. , White, J. B. , & Jones, M. T. (2021). External and internal load measures during preseason training in men collegiate soccer athletes. Journal of Strength and Conditioning Research, 35(9), 2572–2578. 10.1519/JSC.0000000000004092 34431484

[phy270357-bib-0017] Figueiredo, D. H. , Figueiredo, D. H. , Moreira, A. , Goncąlves, H. R. , & Stanganelli, L. C. R. (2019). Effect of overload and tapering on individual heart rate variability, stress tolerance, and intermittent running performance in soccer players during a preseason. Journal of Strength and Conditioning Research, 33(5), 1222–1231. 10.1519/JSC.0000000000003127 30908376

[phy270357-bib-0018] Flatt, A. A. , & Esco, M. R. (2015). Smartphone‐derived heart‐rate variability and training load in a women's soccer team. International Journal of Sports Physiology and Performance, 10(8), 994–1000. 10.1123/ijspp.2014-0556 25756657

[phy270357-bib-0019] Flatt, A. A. , & Esco, M. R. (2016). Evaluating individual training adaptation with smartphone‐derived heart rate variability in a collegiate female soccer team. Journal of Strength and Conditioning Research, 30(2), 378–385. 10.1519/JSC.0000000000001095 26200192

[phy270357-bib-0020] Flatt, A. A. , Esco, M. R. , & Nakamura, F. Y. (2017). Individual heart rate variability responses to preseason training in high level female soccer players. Journal of Strength and Conditioning Research, 31(2), 531–538. 10.1519/JSC.0000000000001482 27227794

[phy270357-bib-0021] Flatt, A. A. , Esco, M. R. , Nakamura, F. Y. , & Plews, D. J. (2017). Interpreting daily heart rate variability changes in collegiate female soccer players. Journal of Sports Medicine and Physical Fitness, 57(6), 907–915. 10.23736/S0022-4707.16.06322-2 26997322

[phy270357-bib-0022] Gilgen‐Ammann, R. , Schweizer, T. , & Wyss, T. (2019). RR interval signal quality of a heart rate monitor and an ECG Holter at rest and during exercise. European Journal of Applied Physiology, 119(7), 1525–1532. 10.1007/S00421-019-04142-5 31004219

[phy270357-bib-0023] Hernando, D. , Hernando, A. , Casajús, J. A. , Laguna, P. , Garatachea, N. , & Bailón, R. (2018). Methodological framework for heart rate variability analysis during exercise: Application to running and cycling stress testing. Medical & Biological Engineering & Computing, 56(5), 781–794. 10.1007/S11517-017-1724-9 28948522

[phy270357-bib-0024] Immanuel, S. , Teferra, M. N. , Baumert, M. , & Bidargaddi, N. (2023). Heart rate variability for evaluating psychological stress changes in healthy adults: A scoping review. Neuropsychobiology, 82(4), 187–202. 10.1159/000530376 37290411 PMC10614455

[phy270357-bib-0025] Jiménez Morgan, S. , & Molina Mora, J. A. (2017). Effect of heart rate variability biofeedback on sport performance, a systematic review. Applied Psychophysiology and Biofeedback, 42(3), 235–245. 10.1007/S10484-017-9364-2 28573597

[phy270357-bib-0026] Julian, R. , Page, R. M. , & Harper, L. D. (2021). The effect of fixture congestion on performance during professional male soccer match‐play: A systematic critical review with meta‐analysis. Sports Medicine, 51(2), 255–273. 10.1007/S40279-020-01359-9 33068272 PMC7846542

[phy270357-bib-0027] Kajaia, T. , Maskhulia, L. , Chelidze, K. , Akhalkatsi, V. , & Kakhabrishvili, Z. (2017). The effects of non‐functional overreaching and overtraining on autonomic nervous system function in highly trained athletes. Georgian Medical News, 264, 97–103. 28480859

[phy270357-bib-0028] Khandoker, A. H. , Jelinek, H. F. , & Palaniswami, M. (2008). Heart rate variability and complexity in people with diabetes associated cardiac autonomic neuropathy. Annual International Conference of the IEEE Engineering in Medicine and Biology Society, 2008, 4696–4699. 10.1109/IEMBS.2008.4650261 19163764

[phy270357-bib-0029] Koenig, J. , & Thayer, J. F. (2016). Sex differences in healthy human heart rate variability: A meta‐analysis. Neuroscience and Biobehavioral Reviews, 64, 288–310. 10.1016/J.NEUBIOREV.2016.03.007 26964804

[phy270357-bib-0030] Malagù, M. , Vitali, F. , Rizzo, U. , Brieda, A. , Zucchetti, O. , Verardi, F. M. , Guardigli, G. , & Bertini, M. (2021). Heart rate variability relates with competition performance in professional soccer players. Heart, 2(1), 36–44. 10.3390/HEARTS2010004

[phy270357-bib-0031] Marcelo de Queiroz Miranda, J. , Luksevicius Rica, R. , & Alcântara Barbosa, W. (2019). Effects of a competitive season on autonomic heart rate modulation in field soccer athletes . http://www.intjexersci.com

[phy270357-bib-0032] McKinney, J. , Velghe, J. , Fee, J. , Isserow, S. , & Drezner, J. A. (2019). Defining athletes and exercisers. The American Journal of Cardiology, 123(3), 532–535. 10.1016/J.AMJCARD.2018.11.001 30503799

[phy270357-bib-0033] Meeusen, R. , Duclos, M. , Foster, C. , Fry, A. , Gleeson, M. , Nieman, D. , Raglin, J. , Rietjens, G. , Steinacker, J. , Urhausen, A. , European College of Sport Science , & American College of Sports Medicine . (2013). Prevention, diagnosis, and treatment of the overtraining syndrome: Joint consensus statement of the European College of Sport Science and the American College of Sports Medicine. Medicine and Science in Sports and Exercise, 45(1), 186–205. 10.1249/MSS.0B013E318279A10A 23247672

[phy270357-bib-0034] Michael, S. , Graham, K. S. , & Oam, G. M. D. (2017). Cardiac autonomic responses during exercise and post‐exercise recovery using heart rate variability and systolic time intervals‐a review. Frontiers in Physiology, 8(May), 259883. 10.3389/FPHYS.2017.00301/BIBTEX PMC5447093 28611675

[phy270357-bib-0035] Miguel, M. , Oliveira, R. , Loureiro, N. , García‐Rubio, J. , & Ibáñez, S. J. (2021). Load measures in training/match monitoring in soccer: A systematic review. International Journal of Environmental Research and Public Health, 18(5), 1–26. 10.3390/IJERPH18052721 PMC7967450 33800275

[phy270357-bib-0036] Mirto, M. , Filipas, L. , Altini, M. , Codella, R. , & Meloni, A. (2024). Heart rate variability in professional and semiprofessional soccer: A scoping review. Scandinavian Journal of Medicine & Science in Sports, 34(6), e14673. 10.1111/SMS.14673 38859758

[phy270357-bib-0037] Moola, S. , Munn, Z. , Tufunaru, C. , Aromataris, E. , Sears, K. , Sfetc, R. , Currie, M. , Lisy, K. , Qureshi, R. , Mattis, P. , & Mu, P. F. (2024). Systematic reviews of aetiology and risk. In E. Aromataris , C. Lockwood , K. Porritt , B. Pilla , & Z. Jordan (Eds.), JBI manual for evidence synthesis. JBI. 10.46658/JBIMES-24-06

[phy270357-bib-0038] Morales, J. , Álamo, J. M. , García‐Masso, X. , Buscà, B. , López, J. L. , Serra‐Añó, P. , & González, L.‐M. (2014). Use of heart rate variability in monitoring stress and recovery in judo athletes. Journal of Strength and Conditioning Research, 28(7), 1896–1905. 10.1519/JSC.0000000000000328 24276307

[phy270357-bib-0039] Morales, J. , Roman, V. , Yáñez, A. , Solana‐Tramunt, M. , Álamo, J. , & Fíguls, A. (2019). Physiological and psychological changes at the end of the soccer season in elite female athletes. Journal of Human Kinetics, 66(1), 99–109. 10.2478/hukin-2018-0051 30988844 PMC6458576

[phy270357-bib-0040] Oliver, M. D. , Baldwin, D. R. , & Datta, S. (2020). The relationship between sleep and autonomic health. Journal of American College Health, 68(5), 550–556. 10.1080/07448481.2019.1583652 30856085 PMC7278032

[phy270357-bib-0041] Patel, P. N. , & Zwibel, H. (2024). Physiology, exercise. StatPearls. https://www.ncbi.nlm.nih.gov/books/NBK482280/ 29489294

[phy270357-bib-0042] Pavlov, V. A. , & Tracey, K. J. (2012). The vagus nerve and the inflammatory reflex—Linking immunity and metabolism. Nature Reviews. Endocrinology, 8(12), 743–754. 10.1038/NRENDO.2012.189 PMC4082307 23169440

[phy270357-bib-0043] Petek, B. J. , Gustus, S. K. , & Wasfy, M. M. (2021). Cardiopulmonary exercise testing in athletes: Expect the unexpected. Current Treatment Options in Cardiovascular Medicine, 23(7), 1–15. 10.1007/S11936-021-00928-Z PMC8963739 35356387

[phy270357-bib-0044] Rabbani, A. , Clemente, F. M. , Kargarfard, M. , & Chamari, K. (2019). Match fatigue time‐course assessment over four days: Usefulness of the hooper index and heart rate variability in professional soccer players. Frontiers in Physiology, 10, 109. 10.3389/fphys.2019.00109 30837890 PMC6390199

[phy270357-bib-0045] Rave, G. , Fortrat, J. O. , Dawson, B. , Carre, F. , Dupont, G. , Saeidi, A. , Boullosa, D. , & Zouhal, H. (2018). Heart rate recovery and heart rate variability: Use and relevance in European professional soccer. International Journal of Performance Analysis in Sport, 18(1), 168–183. 10.1080/24748668.2018.1460053

[phy270357-bib-0046] Ravé, G. , Zouhal, H. , Boullosa, D. , Doyle‐Baker, P. K. , Saeidi, A. , Abderrahman, A. B. , & Fortrat, J. O. (2020). Heart rate variability is correlated with perceived physical fitness in elite soccer players. Journal of Human Kinetics, 72(1), 141–150. 10.2478/hukin-2019-0103 32269655 PMC7126242

[phy270357-bib-0047] Santos‐García, D. J. , Serrano, D. R. , Ponce‐Bordón, J. C. , & Nobari, H. (2022). Monitoring heart rate variability and its association with high‐intensity running, psychometric status, and training load in elite female soccer players during match weeks. Sustainability (Switzerland), 14(22), 14815. 10.3390/su142214815

[phy270357-bib-0048] Santos‐Hiss, M. D. B. , Melo, R. C. , Neves, V. R. , Hiss, F. C. , Verzola, R. M. M. , Silva, E. , Borghi‐Silva, A. , Porta, A. , Montano, N. , & Catai, A. M. (2011). Effects of progressive exercise during phase I cardiac rehabilitation on the heart rate variability of patients with acute myocardial infarction. Disability and Rehabilitation, 33(10), 835–842. 10.3109/09638288.2010.514016 20809873

[phy270357-bib-0049] Schiweck, C. , Gholamrezaei, A. , Hellyn, M. , Vaessen, T. , Vrieze, E. , & Claes, S. (2022). Exhausted heart rate responses to repeated psychological stress in women with major depressive disorder. Frontiers in Psychiatry, 13, 869608. 10.3389/FPSYT.2022.869608 35509881 PMC9058080

[phy270357-bib-0050] Sekiguchi, Y. , Huggins, R. A. , Curtis, R. M. , Benjamin, C. L. , Adams, W. M. , Looney, D. P. , West, C. A. , & Casa, D. J. (2021). Relationship between heart rate variability and acute:Chronic load ratio throughout a season in NCAA D1 men's soccer players. Journal of Strength and Conditioning Research, 35(4), 1103–1109. 10.1519/JSC.0000000000002853 30289866

[phy270357-bib-0051] Shaffer, F. , & Ginsberg, J. P. (2017). An overview of heart rate variability Metrics and norms. Frontiers in Public Health, 5, 258. 10.3389/FPUBH.2017.00258 29034226 PMC5624990

[phy270357-bib-0052] Stein, P. K. , & Pu, Y. (2012). Heart rate variability, sleep and sleep disorders. Sleep Medicine Reviews, 16(1), 47–66. 10.1016/J.SMRV.2011.02.005 21658979

[phy270357-bib-0053] Stølen, T. , Chamari, K. , Castagna, C. , & Wisløff, U. (2005). Physiology of soccer: An update. Sports Medicine, 35(6), 501–536. 10.2165/00007256-200535060-00004 15974635

[phy270357-bib-0054] Thorpe, R. T. , Strudwick, A. J. , Buchheit, M. , Atkinson, G. , Drust, B. , & Gregson, W. (2015). Monitoring fatigue during the in‐season competitive phase in elite soccer players. International Journal of Sports Physiology and Performance, 10(8), 958–964. 10.1123/ijspp.2015-0004 25710257

[phy270357-bib-0055] Thorpe, R. T. , Strudwick, A. J. , Buchheit, M. , Atkinson, G. , Drust, B. , & Gregson, W. (2016). Tracking morning fatigue status across in‐season training weeks in elite soccer players. International Journal of Sports Physiology and Performance, 11(7), 947–952. 10.1123/ijspp.2015-0490 26816390

[phy270357-bib-0056] Thorpe, R. T. , Strudwick, A. J. , Buchheit, M. , Atkinson, G. , Drust, B. , & Gregson, W. (2017). The influence of changes in acute training load on daily sensitivity of morning‐measured fatigue variables in elite soccer players. International Journal of Sports Physiology and Performance, 12, 107–113. 10.1123/ijspp.2016-0433 27918668

[phy270357-bib-0057] Voss, A. , Schroeder, R. , Heitmann, A. , Peters, A. , & Perz, S. (2015). Short‐term heart rate variability—Influence of gender and age in healthy subjects. PLoS One, 10(3), e0118308. 10.1371/JOURNAL.PONE.0118308 25822720 PMC4378923

[phy270357-bib-0058] Voss, B. A. , Schulz, S. , Schroeder, R. , Baumert, M. , & Caminal, P. (2008). Methods derived from nonlinear dynamics for analysing heart rate variability. Philosophical Transactions of the Royal Society A: Mathematical, Physical and Engineering Sciences, 367(1887), 277–296. 10.1098/RSTA.2008.0232 18977726

[phy270357-bib-0059] Weakley, J. , Halson, S. L. , & Mujika, I. (2022). Overtraining syndrome symptoms and diagnosis in athletes: Where is the research? A systematic review. International Journal of Sports Physiology and Performance, 17(5), 675–681. 10.1123/IJSPP.2021-0448 35320774

